# ADAMTS-1 and syndecan-4 intersect in the regulation of cell migration and angiogenesis

**DOI:** 10.1242/jcs.235762

**Published:** 2020-04-08

**Authors:** Jordi Lambert, Kate Makin, Sophia Akbareian, Robert Johnson, Abdullah A. A. Alghamdi, Stephen D. Robinson, Dylan R. Edwards

**Affiliations:** 1 School of Biological Sciences, University of East Anglia, Norwich Research Park, Norwich, NR4 7TJ, UK; 2 Faculty of Medicine and Health Sciences, University of East Anglia, Norwich Research Park, Norwich, NR4 7TJ, UK; 3 Gut Microbes and Health, Quadram Institute Bioscience, Norwich Research Park, Norwich, NR4 7UQ, UK

**Keywords:** ADAMTS, Angiogenesis, Extracellular matrix, Migration

## Abstract

ADAMTS-1 is an extracellular protease with critical roles in organogenesis and angiogenesis. Here we demonstrate a functional convergence of ADAMTS-1 and the transmembrane heparan sulfate proteoglycan syndecan-4 in influencing adhesion, migration and angiogenesis. Knockdown of ADAMTS-1 in endothelial cells resulted in a parallel reduction in cell surface syndecan-4, attributable to increased matrix metalloproteinase-9 (MMP9) activity. Knockdown of either ADAMTS-1 or syndecan-4 increased cellular responses to vascular endothelial growth factor A isoform VEGFA_164_, and increased *ex vivo* aortic ring microvessel sprouting. On fibronectin, knockdown of either protein enhanced migration and promoted formation of long α5 integrin-containing fibrillar adhesions. However, integrin α5 null cells still showed increased migration in response to ADAMTS-1 and syndecan-4 siRNA treatment. Plating of naïve endothelial cells on cell-conditioned matrix from ADAMTS-1 and syndecan-4 knockdown cells demonstrated that the altered adhesive behaviour was matrix dependent, and this correlated with a lack of expression of fibulin-1: an extracellular matrix co-factor for ADAMTS-1 that is known to inhibit migration. These findings support the notion that ADAMTS-1 and syndecan-4 are functionally interconnected in regulating cell migration and angiogenesis, via collaboration with MMP9 and fibulin-1.

This article has an associated First Person interview with the first author of the paper.

## Abstract (precis)

**Summary:** ADAMTS-1 and syndecan-4 collaborate to regulate cell adhesion, migration and integrin α5 trafficking, and to sequester VEGFA_164_, inhibiting angiogenesis.

## INTRODUCTION

The ADAMTS (a disintegrin and metalloproteinase with thrombospondin motifs) family of extracellular proteases includes 19 members in humans, with diverse roles in tissue development and homeostasis (Kuno et al., 1997; Porter et al., 2005). Their essential functions are underlined by the recognition that several family members are encoded by genes that are responsible for inherited genetic disorders when mutated, while others are associated with pathologies including cancer, arthritis and cardiovascular disease when aberrantly expressed (Hubmacher and Apte, 2015; Kelwick et al., 2015b; Mead and Apte, 2018).

The ADAMTSs are zinc-dependent metalloproteinases with a compound domain structure, each possessing a catalytic domain containing the metalloproteinase and disintegrin-like features, followed by a modular ancillary domain that differs between subgroups of family members and is important for their biological actions (Kuno et al., 1997; Porter et al., 2005). The largest clade in the ADAMTS family are identified as proteoglycanases that can cleave a variety of proteoglycans, including versican, aggrecan and brevican, as well as other extracellular matrix proteins (Hubmacher and Apte, 2015; Kelwick et al., 2015b). First discovered in 1997, ADAMTS-1 is the prototype of the family and a member of the proteoglycanase clade that also includes ADAMTS-4, -5, -8, -9, -15 and -20. The ability of ADAMTS-1 to cleave structural extracellular matrix (ECM) components is physiologically relevant as demonstrated by *Adamts1−/−* knockout mice, which exhibit abnormally high rates of perinatal lethality due to multiple organ defects, in particular severe kidney malformation and cardiac defects (De Arao Tan et al., 2013; Krampert et al., 2005). The surviving female mice suffer from infertility, due to the ineffective cleavage of versican during ovarian maturation (Krampert et al., 2005; Mittaz et al., 2004; Shindo et al., 2000).

However, as well as its proteolytic function, ADAMTS-1 also interacts with other proteins including latent TGF-β (Bourd-Boittin et al., 2011) and fibulin-1, which acts as a co-factor (Lee et al., 2005). ADAMTS-1 has many context-dependent effects in biological processes such as migration, invasion and cell signalling, which are relevant to its impact on physiology and pathophysiology, indicating it acts through multiple mechanisms (De Arao Tan et al., 2013). This is reflected in its anti-angiogenic actions, which involve both proteolytic and non-proteolytic mechanisms, the former by mediating the release of highly anti-angiogenic fragments of thrombospondin (TSP)-1 and -2 (Gustavsson et al., 2010; Lee et al., 2006) and the latter via direct binding and sequestration of the vascular endothelial growth factor isoform VEGFA_165_ (Fu et al., 2011; Luque et al., 2003).

Another significant proteoglycan partner of ADAMTS-1 is syndecan-4 (Rodríguez-Manzaneque et al., 2009). Syndecan-4 is a ubiquitously expressed heparan sulfate proteoglycan that acts as a key mediator of several cellular processes including adhesion, proliferation and endocytosis (Couchman and Woods, 1999; Elfenbein and Simons, 2013; Elfenbein et al., 2012). Its heparan sulfate glycosaminoglycan (GAG) chains provide binding sites for heparin-binding growth factors such as fibroblast growth factors (FGFs), platelet-derived growth factors (PDGFs) and vascular endothelial growth factors (VEGFs) (Elfenbein and Simons, 2013). The binding of these growth factors to syndecan-4 can have several consequences: activation of cellular signalling can occur through syndecan-4 acting as a co-receptor that presents the growth factor ligand to its signalling receptor, as in the case of FGF, or there can be direct activation of downstream signalling mediated by syndecan-4 itself, such as protein kinase Cα (PKCα) (Oh et al., 1997a,b). In addition, syndecan-4 can regulate growth factor bioavailability by acting as a cell-bound reservoir that can be released by subsequent proteolytic cleavage (Bergers et al., 2000; Ramnath et al., 2014). In addition to its role as a signalling regulator, syndecan-4 is also a key mediator in focal adhesion formation. Fibroblasts from syndecan-4 null mice exhibit impaired adhesion to fibronectin (Ishiguro et al., 2000). Via the binding and activation of PKCα, syndecan-4 facilitates α5β1 integrin binding to its substrate fibronectin, allowing maturation of focal adhesions (Bass et al., 2007; Mostafavi-Pour et al., 2003). Given its key role as a nexus of signalling and adhesion mechanisms, the relative levels and localisation of syndecan-4 are therefore critical determinants of cellular behaviour.

Several reports have connected the actions of ADAMTS enzymes with syndecan-4 (SDC4), including ADAMTS-1 and -4 (Rodríguez-Manzaneque et al., 2009), ADAMTS-5 (Echtermeyer et al., 2009; Wang et al., 2011), ADAMTS-6 and -10 (Cain et al., 2016) and ADAMTS-15 (Kelwick et al., 2015a). In this study, we have uncovered details of a complex inter-relationship between ADAMTS-1 and syndecan-4 in murine fibroblasts and endothelial cells. We have shown that acute depletion of ADAMTS-1 leads to a concomitant reduction in cell surface levels of syndecan-4, such that downregulation of either syndecan-4 or ADAMTS-1 has similar consequences on cell behaviour, shown by increases in cellular migration and striking changes to focal adhesions, both of which were fibronectin dependent. Furthermore, loss of either ADAMTS-1 or syndecan-4 in endothelial cells led to increases in angiogenesis, which we demonstrate is due to reduced ability of cells to sequester the pro-angiogenic growth factor VEGFA_164_. Moreover, these effects have downstream consequences on α5 integrin trafficking, but α5 integrin is not essential for them to occur. These results demonstrate the existence of an interplay between ADAMTS-1 and syndecan-4 that orchestrates cell adhesion, migration and angiogenesis.

## RESULTS

### Knockdown of ADAMTS-1 results in reduction of cell surface syndecan-4

**Fig. 1.**
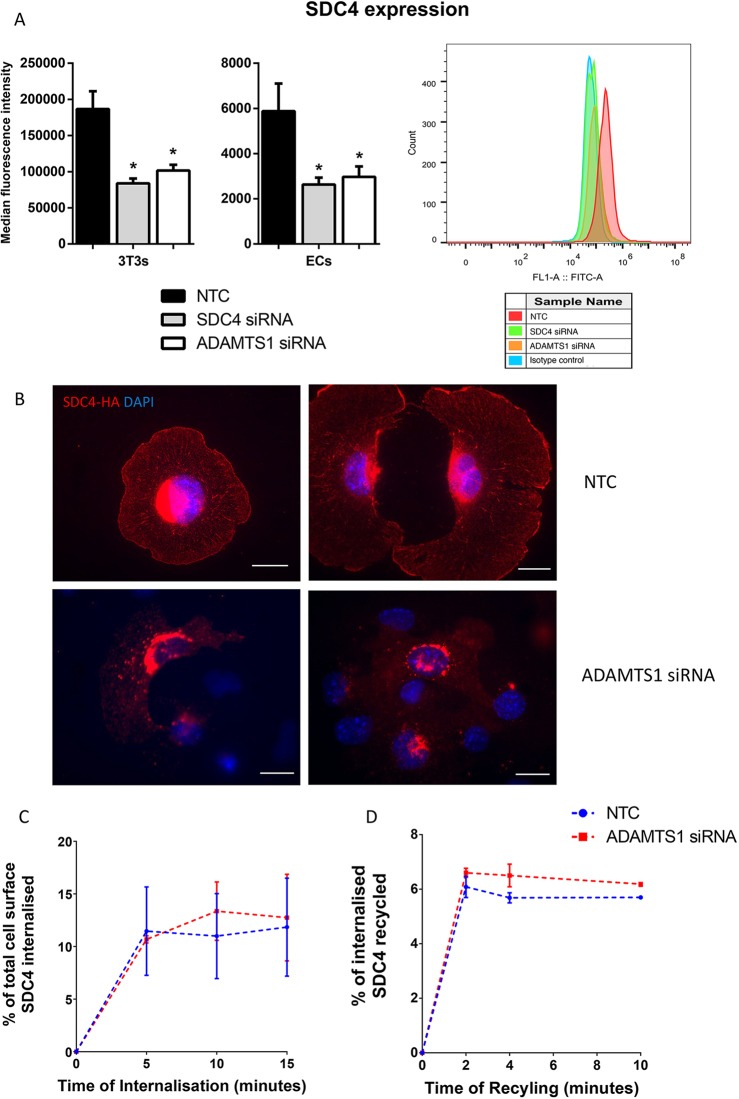
**ADAMTS-1 regulates syndecan-4 expression but not via altered recycling or internalisation.** (A) Flow cytometric analysis of syndecan-4 (SDC4) surface levels on lung microvascular endothelial cells (ECs) and 3T3 fibroblast cells (3T3s) treated with non-targeting control (NTC), SDC4 or ADAMTS-1 siRNA. The graph (far right) is a representative histogram showing median fluorescence intensity. Values were calculated after gating on forward and side scatter and normalised to isotype controls; *n*=3 biologically independent experiments. Charts show means±s.e.m., *P<0.05 compared with control cells by Student's *t*-test. (B) Representative image of HA-tag (red) and DAPI (blue) immunostained ECs expressing HA-tagged SDC4 treated with siRNA against ADAMTS-1 or NTC on fibronectin; *n*=3 independent experiments. Scale bars, 20 µm. (C) SDC4 internalisation assay; EC cell surface proteins were biotinylated using a cleavable, membrane non-permeable biotin. ECs were incubated to allow biotinylated proteins to internalise for indicated time intervals. Following this, cell surface biotin was removed using the reducing agent MesNa, leaving only membrane proteins that had been internalised and biotinylated. ECs were lysed and protein was collected prior to internalisation, and at 5, 10 and 15 min of internalisation. An SDC4 capture ELISA was performed on lysed ECs. Streptavidin was used to detect biotinylated SDC4. Graphs indicate the percentage of internalised biotinylated syndecan-4, relative to starting level of cell surface syndecan-4 (*n*=3, bars represent s.e.m.). (D) SDC4 recycling assay; ECs were cell surface biotinylated, and membrane proteins were then allowed to internalise for 20 min, followed by MesNa treatment, to remove cell surface biotin. After the MesNa treatment, internalised biotin was allowed to return to the cell surface. Biotinylated protein that had returned to the surface was again removed using MesNa and an ELISA was performed as in panel C. Values represent percentage of recycled syndecan-4 relative to total internalised after 20 min (*n*=3 independent experiments, bars represent s.e.m.).

We have used small interfering RNA (siRNA) to deplete either ADAMTS-1 or syndecan-4 in murine 3T3 fibroblast cells (3T3s) and lung microvascular polyoma middle T-antigen immortalised endothelial cells (ECs). mRNA knockdown was confirmed using TaqMan quantitative PCR (qPCR) (Fig. S1). Our initial hypothesis was that as ADAMTS-1 has been reported to cleave the N-terminal domain of syndecan-4, depletion of ADAMTS-1 would result in accumulation of syndecan-4 on the cell surface (Rodríguez-Manzaneque et al., 2009). However, flow cytometric analysis revealed that siRNA knockdown of ADAMTS-1 resulted in a significant concomitant decrease in cell surface syndecan-4, equivalent to that seen with syndecan-4 knockdown (Fig. 1A). To confirm this visually, immunocytochemistry was performed; however, as in our hands staining using commercially available syndecan-4 antibody was unsuccessful, cells were transfected with a HA-tagged syndecan-4 construct (HA-SDC4). This confirmed reduced cell surface HA-SDC4 display in ADAMTS-1-depleted cells when compared with cells treated with non-targeting control siRNA (Fig. 1B). To confirm that the interaction was specific to ADAMTS-1 and syndecan-4, levels of other endothelial expressed syndecans were recorded following ADAMTS-1 and syndecan-4 siRNA depletion. Syndecan-3 was not expressed in ECs; syndecan-1 and -2 were, but the cell surface levels of these proteins were unaffected by ADAMTS-1 or syndecan-4 siRNA depletion (Fig. S2).

To elucidate the mechanism by which ADAMTS-1 regulates syndecan-4 expression, we first investigated whether knockdown of ADAMTS-1 led to changes in *Sdc4* gene expression. ADAMTS-1 siRNA did not alter *Sdc4* expression at the RNA level (Fig. S1). Therefore, we hypothesised that loss of cell surface syndecan-4 could be a result of changes in membrane trafficking, so we carried out cell surface biotinylation-based internalisation and recycling assays. ECs were surface labelled with cleavable biotin, incubated for time intervals to allow internalisation, then biotin remaining on the cell surface was cleaved. Syndecan-4 internalisation was quantified by a syndecan-4 capture enzyme-linked immunosorbent assay (ELISA) and biotin detection using streptavidin (Fig. 1C). For the recycling assay cells were incubated further, to allow biotinylated protein to recycle to the surface (Fig. 1D). These experiments demonstrated that neither the rate of syndecan-4 internalisation nor levels of recycling to the membrane were changed as a result of ADAMTS-1 knockdown.

### Loss of cell surface syndecan-4 is MMP9 dependent

**Fig. 2.**
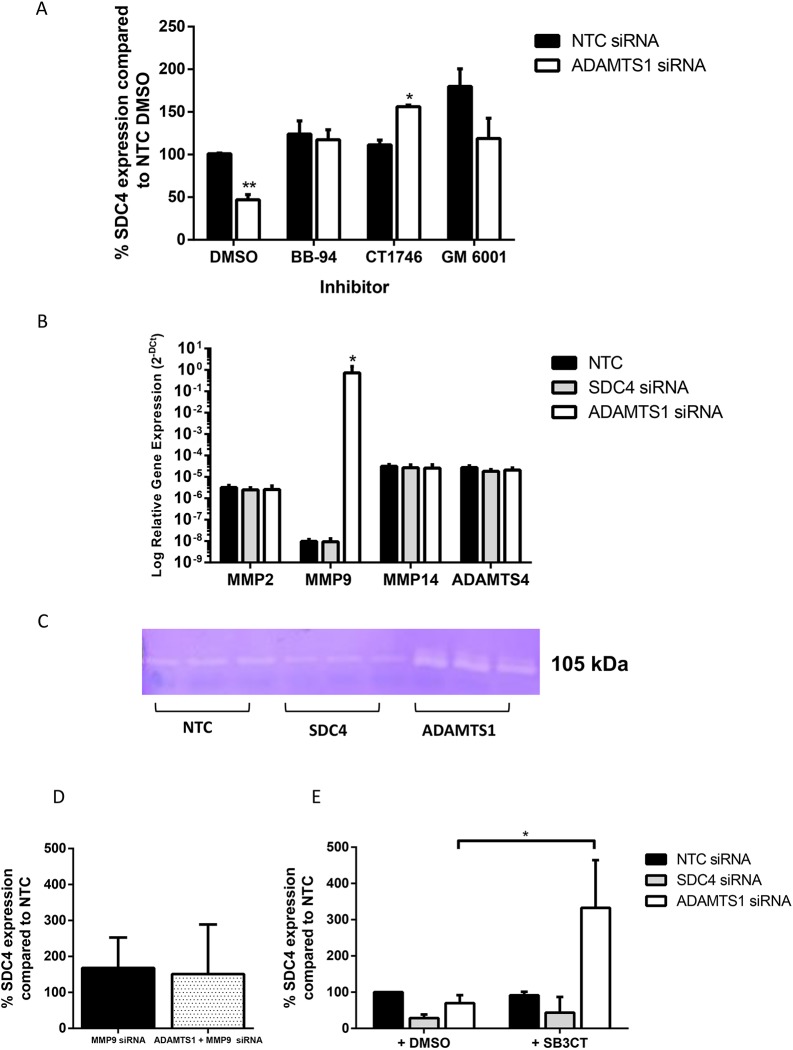
**ADAMTS-1 siRNA-mediated loss of surface SDC4 is dependent on MMP activity.** (A) Flow cytometric analysis of SDC4 cell surface levels. ECs were transfected with NTC or ADAMTS-1 siRNA, then treated with an MMP inhibitor: BB-94, CT1746, GM 6001 or DMSO vehicle control. Bar chart shows percentage SDC4 expression relative to NTC DMSO-treated cells (*n*=3, *P<0.05, **P<0.01, compared with control cells by Student's *t*-test). (B) TaqMan qPCR for MMP2, MMP9, MMP14 and ADAMTS4 on RNA from ECs treated with NTC, SDC4 or ADAMTS1 siRNA. Bar chart shows relative expression, normalised to a housekeeping control (18S) (*n*=4 independent experiments, *P<0.05 compared with control by Student's *t*-test). (C) A representative gelatin zymogram showing MMP9 activity in the conditioned media of siRNA-treated ECs. (D) Flow cytometric analysis of SDC4 surface levels. ECs were transfected with MMP9, or a combination of MMP9 and ADAMTS-1 siRNA. Bar chart shows percentage SDC4 expression relative to NTC siRNA-treated cells (*n*=3 independent experiments). (E) Flow cytometric analysis of SDC4 following NTC, SDC4 or ADAMTS1 siRNA depletion and treatment with MMP9 inhibitor SB-3CT (*n*=3 independent experiments).

As reduced syndecan-4 levels did not appear to be a result of altered transcription or membrane trafficking, we speculated that the decreased cell surface syndecan-4 was a result of increased shedding. As matrix metalloproteinases (MMPs) are known cleavers of syndecans, they were considered as possible mediators of this shedding (Manon-Jensen et al., 2010). ECs were treated with one of the following three broad-spectrum MMP inhibitors: BB-94, CT-1746, GM 6001 or a DMSO vehicle control. Treatment with any of the three MMP inhibitors resulted in accumulation of syndecan-4 at the cell surface, and in cells treated with a combination of an MMP inhibitor and ADAMTS-1 siRNA, syndecan-4 levels were not significantly reduced (Fig. 2A). This suggests that MMPs are responsible for the reduction in cell surface syndecan-4 seen in response to loss of ADAMTS-1.

To identify the specific enzyme responsible, a panel of MMPs known to be expressed in endothelial cells were profiled: MMP2, MMP9, MMP14 (MT1-MMP) and ADAMTS-4 (Bergers et al., 2000; Genís et al., 2006; Hsu et al., 2012)_._ ADAMTS-1 siRNA knockdown resulted in significantly increased expression of *Mmp9* compared with non-targeting control (NTC) or syndecan-4 siRNA-treated cells, which expressed almost no *Mmp9*. In both treated and untreated cells, *Mmp2*, *Mmp14* and *Adamts4* expression was unchanged (Fig. 2B). The increase in *Mmp9* RNA expression was reflected in increased MMP9 activity, as shown by gelatin zymography of conditioned media from ADAMTS-1 siRNA-, syndecan-4 siRNA- and NTC-treated cells (Fig. 2C). In these experiments we were unable to detect syndecan-4 that had been shed from the cell surface into the medium under any conditions, indicating that this is probably rapidly degraded. However, treatment with MMP9 siRNA (Fig. 2D) or MMP9 specific inhibitor SB-3CT (Fig. 2E) was sufficient to reverse the phenotype resulting from ADAMTS-1 knockdown. This demonstrates that increased expression of MMP9, known to cleave syndecan-4, is directly responsible for the reduced syndecan-4 levels seen after ADAMTS-1 siRNA treatment (Ramnath et al., 2014; Reine et al., 2019).

### Syndecan-4 contributes to sequestration of VEGFA_165_ by ADAMTS-1

ADAMTS-1 acts as an inhibitor of angiogenesis by sequestering VEGFA_164_, a key pro-angiogenic factor. This sequestration prevents VEGF from binding and activating its major receptor VEGFR2 (Luque et al., 2003). ADAMTS-1 exclusively binds VEGFA_164_, and the interaction is dependent on the heparin-binding domain. Previous work by Iruela-Arispe et al. has suggested that a heparin sulfate proteoglycan such as a member of the syndecan family may provide a bridge to facilitate this interaction (Iruela-Arispe et al., 2003). Alternatively, syndecan-4 may function as co-receptor for VEGF in an interaction similar to that reported for FGF2; however, although syndecan-2 endothelial specific deletion in mice leads to arteriogenic defects and impaired VEGF signalling, no such abnormalities are observed in mice with a deleted syndecan-4 (Corti et al., 2019; Horowitz et al., 2002).

**Fig. 3.**
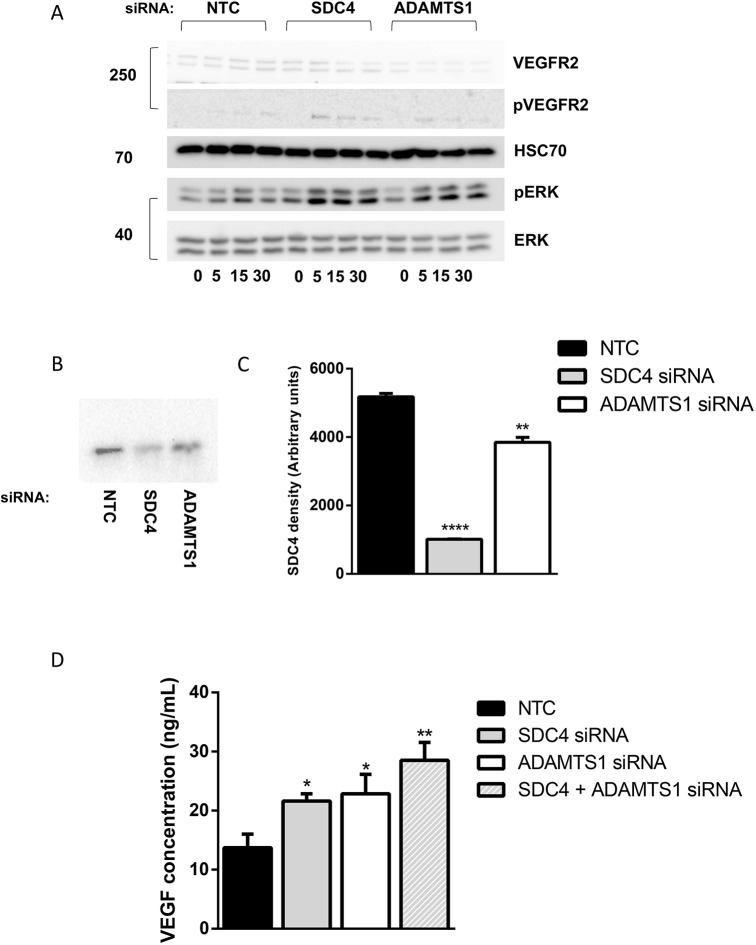
**SDC4 contributes to the sequestration of VEGFA_164_ by ADAMTS-1.** (A) VEGF signalling time course. siRNA-treated ECs were adhered overnight on fibronectin. Serum-starved cells were stimulated with 30 ng/ml VEGF for 0, 5, 15 and 30 min. Western blots were performed on lysates with anti-VEGFR2, anti-ERK, and their phosphorylated forms. HSC70 was used as a loading control. Blot is representative of four independent experiments. (B) Western blot using an anti-SDC4 antibody on fractions from VEGF immunoprecipitations (IPs) carried out on HUVECs with lentiviral silencing targeting NTC, ADAMTS-1 or SDC4. (C) ImageJ densitometric quantification of IP western blots (*n*=3 independent experiments, charts show means±s.e.m., **P<0.01, ****P<0.0001 compared with control cells by Student's *t*-test). (D) VEGF sequestration ELISA on ECs treated with NTC, SDC4 or ADAMTS-1 siRNA. Cells were kept at 4°C, incubated in serum-free medium containing 30 ng/ml VEGFA_164_ for 30 min. Medium was collected and a VEGF sandwich ELISA was performed to detect unbound VEGF (*n*=3 independent experiments, bars represent s.e.m., *P<0.05, **P<0.01 compared with control cells by Student's *t*-test).

To determine if syndecan-4 is capable of binding VEGFA_164_, co-immunoprecipitations were performed. Immunoprecipitation (IP) of VEGFA_164_ was able to co-precipitate syndecan-4, revealing that syndecan-4 either directly or indirectly binds VEGF. When cells were treated with ADAMTS-1 siRNA, less syndecan-4 was found upon co-precipitation (Fig. 3B,C). The reduction in syndecan-4 seen with ADAMTS-1 siRNA may be an indicator that the interaction involves ADAMTS-1; however, it may also be a result of the reduced cell surface syndecan-4 seen with ADAMTS-1 siRNA treatment.

We next sought to establish if syndecan-4 supported signalling, or aided in the sequestration of VEGF by ADAMTS-1, by examining impact of ADAMTS-1 or syndecan-4 siRNA on VEGF signalling. Endothelial cells were serum-starved for 3 h, then stimulated with 30 ng/ml VEGFA_164_ (the mouse equivalent of human VEGFA_165_). Cells were lysed at 0, 5, 15 and 30 min post-stimulation, and lysates collected were run on a western blot to look for changes in the VEGF signalling pathway. Upon siRNA knockdown of either ADAMTS-1 or syndecan-4, increased phospho-VEGFR2 and phospho-extracellular signal-related kinase (ERK) were seen, indicating an increase in cellular responsiveness to exogenous VEGFA_165_ when ADAMTS-1 or syndecan-4 levels are reduced (Fig. 3A; Fig. S3).

These data suggest that syndecan-4 binds and sequesters VEGFA_165_ alongside ADAMTS-1; this hypothesis was further investigated using a VEGF ELISA to assess how much VEGF cells were able to sequester. As before, 30 ng/ml VEGFA_164_ was added to ECs cooled to 4°C to prevent signalling. A western blot was performed to confirm that signalling pathways were not activated (Fig. S4A), and qPCR confirmed *Vegfa* mRNA levels were unchanged (Fig. S4B). The cells were kept at 4°C for 30 min to allow binding. The medium was then collected and an ELISA was performed to quantify the amount of unbound VEGF remaining in the medium. When cells were treated with ADAMTS-1, syndecan-4 or both siRNAs, the amount of free VEGF remaining in the medium significantly increased (Fig. 3D). Taken together, these data suggest that the loss of ADAMTS-1 or syndecan-4 prevents the sequestration of VEGF, leaving it freely available to activate downstream signalling pathways.

### ADAMTS-1 and syndecan-4 sequestration of VEGF inhibits angiogenesis

**Fig. 4.**
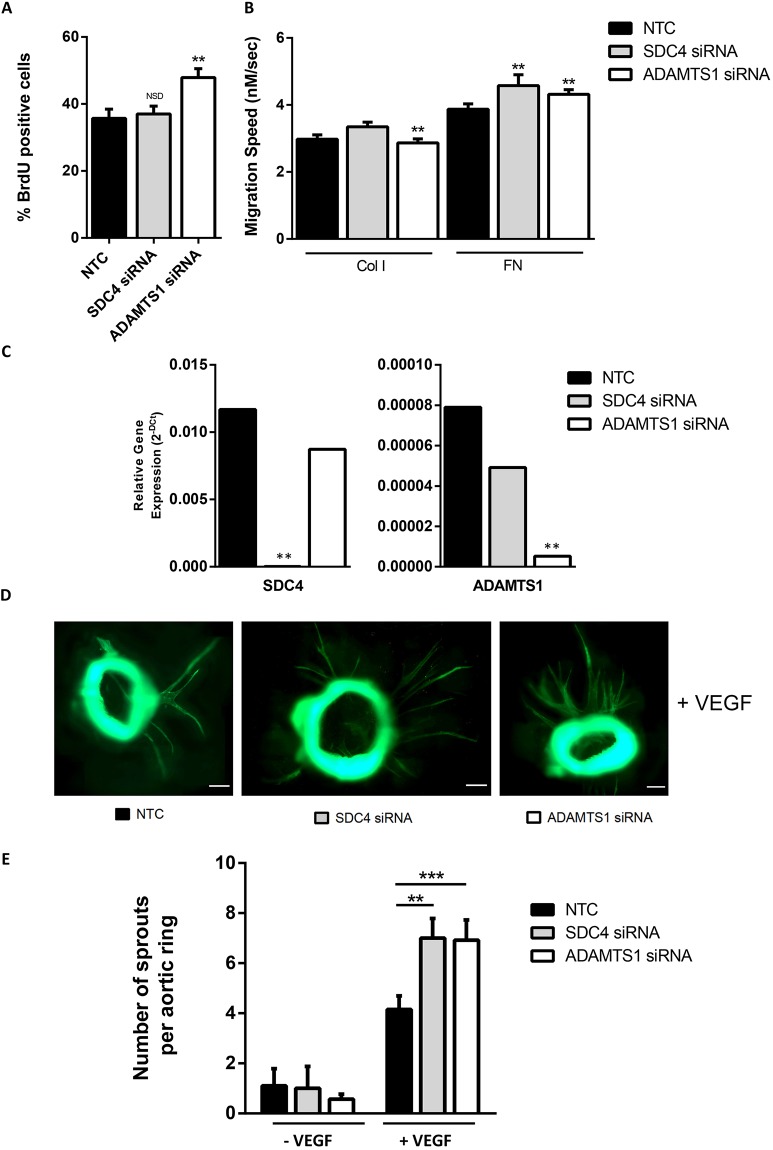
**ADAMTS-1 and SDC4 siRNA treatment results in increased angiogenesis.** (A) Proliferation assay on siRNA-treated ECs allowed incorporation of BrdU for 12 h. Cells were fixed and stained for BrdU and DAPI. Total number of nuclei and BrdU-positive nuclei were counted; bar chart shows the percentage of proliferating cells (*n*=4; **P<0.01; NSD, no significant difference). (B) 2D migration assay on siRNA-treated ECs seeded onto 10 µg/ml collagen I or fibronectin matrix and allowed to adhere overnight. Migration velocity was determined with time-lapse microscopy over a period of 16 h (*n*≥100 cells in four independent experiments, charts show means±s.e.m., **P<0.001). (C) TaqMan qPCR of aortic rings treated with ADAMTS1 or SDC4 siRNA showing relative levels of SDC4 and ADAMTS-1 in three independent experiments; bars represent s.e.m., **P<0.001 compared with control by Student's *t*-test**.** (D) Microvessel sprouting of aortic ring explants from 6- to 8-week-old mice treated with indicated siRNA. Aortae were harvested from wild-type C56BL/6 mice in four independent experiments (number of mice per experiments: 10, 8, 10 and 7). Aortae were stripped and cut into rings that were equally distributed between conditions. Representative images of aortic rings stained with fluorescein isothiocyanate (FITC)-conjugated BS1-lectin are shown. Images were obtained on an epifluorescence microscope, and several images were stitched in Fiji to give the complete field of view, followed by filtering to enhance. Scale bars, 200 µm. (E) Bar chart showing the total number of microvessel sprouts per aortic ring, 6 days post-VEGF stimulation (*n*≥50 rings per condition, *n*=4 independent experiments of 10, 8, 10 and 7 mice. Bar charts show means±s.e.m., **P<0.001, ***P<0.0001 compared with control rings by Student's *t*-test).

The physiological relevance of the ability of ADAMTS-1 and syndecan-4 to sequester VEGF was investigated. The process of angiogenesis requires both proliferation and migration of endothelial cells, which can be stimulated by VEGF. Bromodeoxyuridine (BrdU) labelling of siRNA-treated cells revealed increased proliferation in ADAMTS-1-depleted cells, but not syndecan-4-depleted cells (Fig. 4A). As syndecan-4 is known to interact with numerous other growth factors (FGFs, EGFs, PDGFs) this may be a result of multiple pathway involvement (Harburger and Calderwood, 2008).

As conflicting reports exist with regard to the roles of ADAMTS-1 and syndecan-4 in cell migration (Bass et al., 2007; Krampert et al., 2005; Obika et al., 2012; Rodríguez-Manzaneque et al., 2009), random migration assays were carried out on siRNA-depleted cells to assess the effects of their knockdown on motility. siRNA-treated endothelial cells were plated on either 10 µg/ml collagen I or 10 µg/ml fibronectin, and time-lapse microscopy was performed over a period of 16 h to track cell migration. Migration speed was significantly increased with both ADAMTS-1 and syndecan-4 on fibronectin matrices only (Fig. 4B).

To see if the altered proliferation and migration resulted in a corresponding increase in angiogenesis, the mouse aortic ring assay was utilised. Aortae were harvested from 6- to 8-week-old mice, sliced into rings and treated with siRNA against ADAMTS-1 or syndecan-4. The rings were embedded in matrix, and the number of sprouts that formed was used as a marker of angiogenesis (Baker et al., 2012). Both siRNAs successfully depleted their targets in the aortic rings (Fig. 4C). Both ADAMTS-1 and syndecan-4 depletion resulted in a marked increase in VEGF-dependent new vessel sprouting (Fig. 4D,E), demonstrating a physiologically relevant effect of the sequestration of VEGF by ADAMTS-1 and syndecan-4.

### Adhesions are altered in ADAMTS-1 and syndecan-4 siRNA cells on fibronectin matrices

**Fig. 5.**
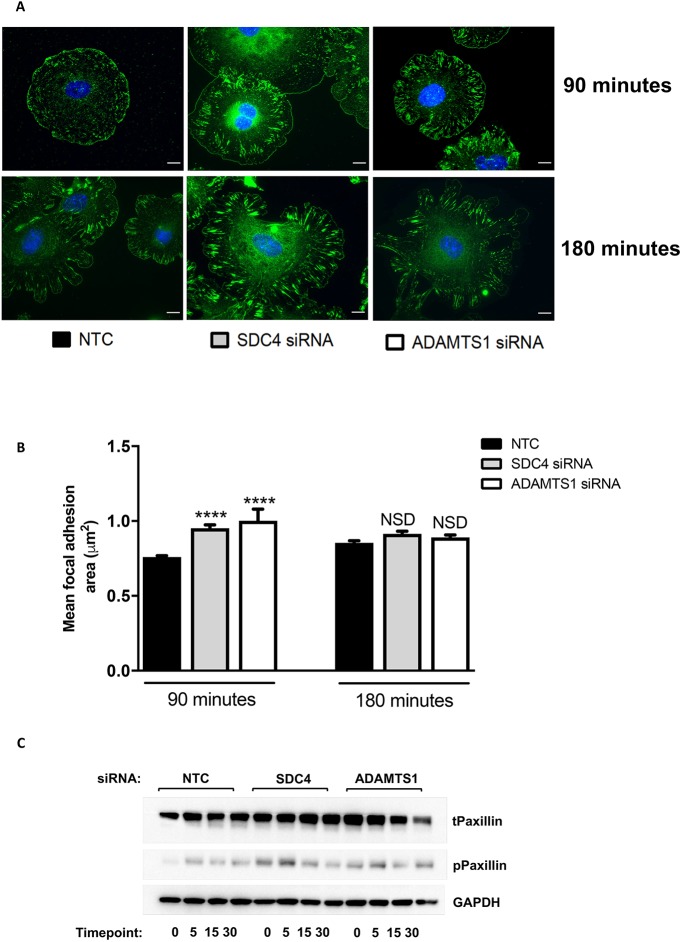
**siRNA depletion of ADAMTS-1 or SDC4 results in altered focal adhesions.** (A) Representative images of siRNA-treated ECs adhered to fibronectin-coated glass coverslips for 90 or 180 min. Cells were fixed and stained for focal adhesions with paxillin (green) and the nucleus with DAPI (blue); *n*=3 independent experiments. Scale bars, 10 µm. (B) Area of paxillin positive focal adhesions, calculated using ImageJ. Graph shows average size of adhesions, *n*=20 cells per condition from three independent experiments, means±s.e.m., ****P<0.00001, calculated using a Kruskal–Wallis test. (C) Western blot analysis of paxillin signalling using an anti-phospho-paxillin antibody in siRNA-treated ECs adhered to fibronectin, serum starved for 3 h and then stimulated with 30 ng/ml VEGF for 0, 5, 15 and 30 min. GAPDH was used as a loading control; *n*=3 independent experiments.

As knockdown of either ADAMTS-1 or syndecan-4 led to altered migration speeds on fibronectin but not on collagen I, focal adhesion parameters were assessed on both matrices. Cells were seeded on a collagen I or fibronectin matrix for 90 or 180 min. Staining for paxillin was performed to visually assess focal adhesions, and their size and number were calculated using ImageJ. While on both collagen and fibronectin the numbers of focal adhesions were unchanged in siRNA-depleted cells, adhesions to fibronectin matured more quickly. At 90 min post-adherence to fibronectin, the average focal adhesion size was larger, with a higher percentage of adhesions reaching a more mature stage (Fig. 5A,B). The majority of adhesions remained as small nascent adhesions (<2 µm^2^) (NTC: 95.6%; syndecan-4: 90.9%; ADAMTS-1: 92%); however, a higher percentage of cell adhesions in siRNA-treated cells had developed into focal adhesions (2–6 µm^2^) (NTC: 4.3%; syndecan-4: 8.5%; ADAMTS-1: 7.2%). By 180 min this difference no longer existed, suggesting that the siRNA-treated cells do not form more large adhesions, but rather their adhesions mature at a faster rate. Adhesions to collagen were unaffected (Fig. S5), confirming that the observed effects are due to interaction with the fibronectin matrix. These changes in focal adhesions of ADAMTS-1 or syndecan-4 knockdown cells on fibronectin were also apparent in a marked increase in paxillin signalling in response to VEGF (Fig. 5C; Fig. S6). This supports the proposition that ADAMTS-1 and syndecan-4 act to sequester VEGF, reducing its bioavailability, and that their depletion leads to enhanced VEGF signalling and subsequent changes to focal adhesion morphology.

### α5 Integrin localisation is disrupted in the absence of ADAMTS-1 or syndecan-4

**Fig. 6.**
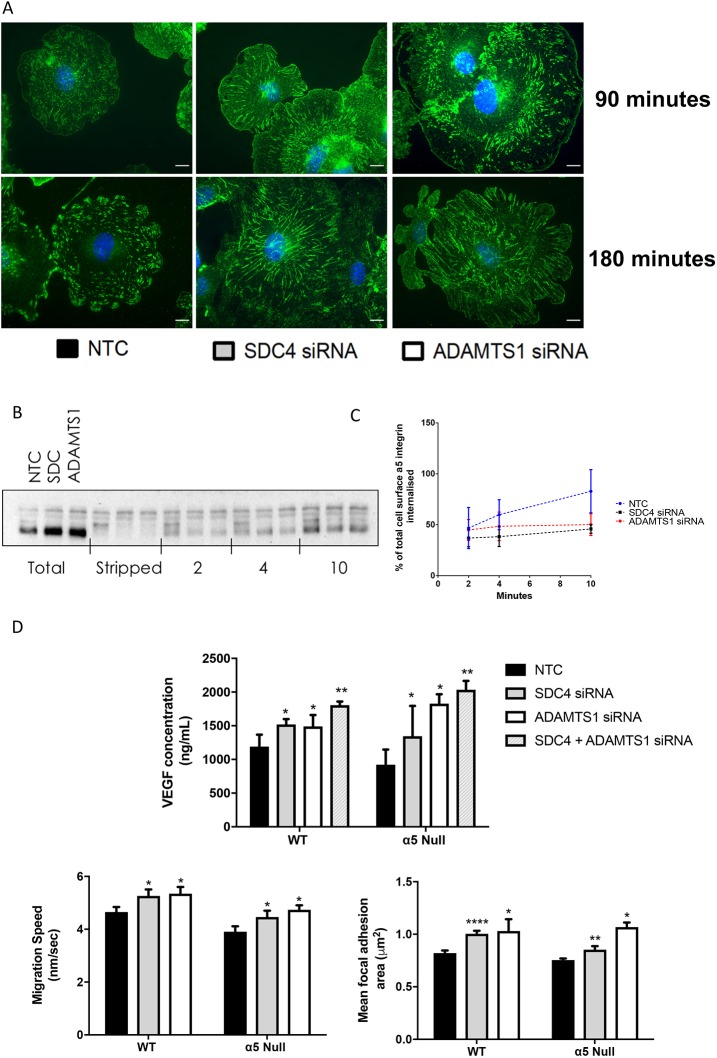
**Depletion of ADAMTS-1 or SDC4 results in downstream alterations to α5 integrin behaviour.** (A) Representative images of siRNA-treated ECs adhered to fibronectin-coated glass coverslips for 90 or 180 min. Cells were fixed and stained for α5 integrin (green) and DAPI (blue); *n*=3 independent experiments. Scale bars, 10 µm. (B) Representative western blot of IP lysates from internalisation assays using an anti-α5 integrin antibody. Cell surface proteins of ECs seeded on fibronectin were biotinylated, and allowed to internalise for 2, 4 and 10 min. Post-internalisation, remaining surface biotin was ‘stripped’ using a membrane-impermeable reducing agent (MesNa). Cells were lysed and immunoprecipitated for biotin. The blot shows ‘total’ surface α5 (from cells not internalised and not stripped), cells not internalised but ‘stripped’ as controls, and α5 internalisation at each time point. (C) Quantification of α5 integrin internalisation assays (*n*=3 independent experiments). (D) Experiments from Figs 3D, 4B and 5B were repeated in ECs isolated from α5 null mice and their wild-type controls (*n*=3 independent experiments, charts show means± s.e.m., *P<0.05, **P<0.01, ****P<0.00001, compared with control cells by Student's *t*-test).

As our data showed perturbation of fibronectin-dependent adhesions following ADAMTS-1 or syndecan-4 depletion, the contribution of α5 integrin (α5), the major fibronectin receptor in nascent focal adhesions in endothelial cells, was considered. Immunocytochemical visualisation of α5 revealed the development of long, fibrillar-like adhesions after 180 min adherence to fibronectin in cells treated with ADAMTS-1 or syndecan-4 siRNA, as opposed to the more traditional α5 localisation in focal adhesions (<6 µm^2^) seen in NTC cells (Fig. 6A).

As syndecan-4 is known to play a role in integrin surface trafficking, biotinylation-based internalisation assays were performed as before, to assess α5 membrane trafficking (Morgan et al., 2013). To quantify internalisation, immunoprecipitation of biotin followed by western blot detection of α5 was carried out. Western blotting revealed an increase in total cell surface α5 in ADAMTS-1 and syndecan-4 siRNA-treated cells, as well as decreased levels of internalisation, suggesting that the altered adhesions may be a result of α5 accumulation due to impaired internalisation (Fig. 6B,C). To test whether this retention of α5 on the cell surface in ADAMTS-1 and syndecan-4 knockdown cells was responsible for the altered cell characteristics, we again performed the experiments above in α5-null endothelial cells treated with ADAMTS-1 or syndecan-4 siRNA. However, although α5-null endothelial cells showed reduced migration compared with their wild-type counterparts, the same phenotypes of increased rates of migration and focal adhesion maturation, and decreased VEGF sequestration following ADAMTS-1 or syndecan-4 knockdown were maintained (Fig. 6D). These data suggest that the changes in α5 integrin are probably a downstream consequence of changes in cell behaviour mediated by ADAMTS-1 and syndecan-4 knockdown, rather than α5 integrin being the key effector of the phenotypes.

### EC behaviour and signalling phenotypes are maintained in response to conditioned matrix

**Fig. 7.**
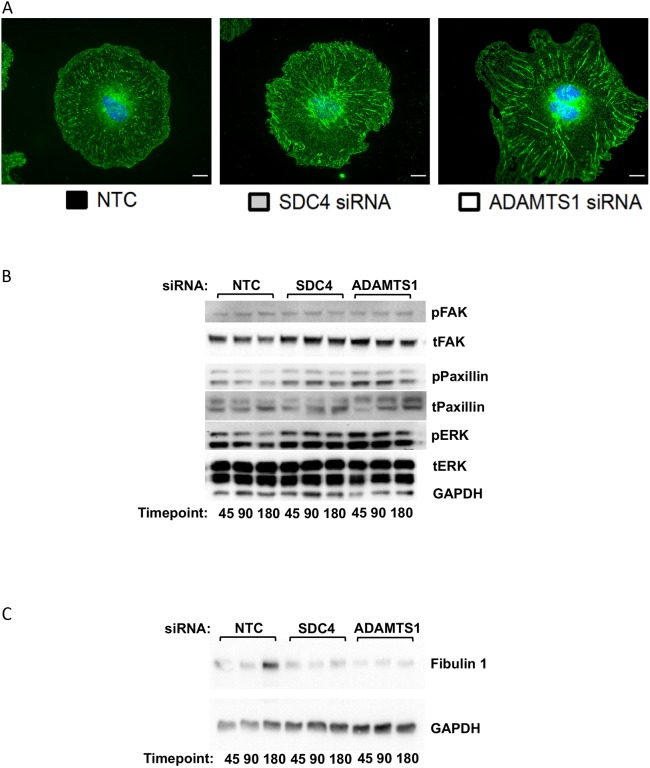
**EC behaviour and signalling phenotypes are maintained in response to conditioned matrix.** (A) Representative images of untreated ECs after 180 min on conditioned matrix (CM) from siRNA-treated ECs allowed to produce matrix for 48 h before being removed. Cells were fixed and stained for α5 integrin (green); *n*=3 independent experiments. Scale bars, 10 µm. (B) Western blot of untreated ECs seeded on CM for 45, 90 and 180 min, using anti-total (t) and phosphorylated (p) FAK, ERK and paxillin. GAPDH was used as a loading control; *n*=3 independent experiments. (C) Western blot for fibulin-1 of siRNA-treated ECs seeded onto fibronectin for 45, 90 or 180 min. GAPDH was used as a loading control. Blot is representative of three independent experiments.

As the mechanism by which ADAMTS-1 and syndecan-4 siRNA increased cell migration and changed focal adhesions was not dependent on integrin α5, modulation of the ECM itself was considered, especially as these responses were reliant on the cells being on a fibronectin matrix. In order to assess this, experiments were performed using ‘conditioned matrix’ (CM) from siRNA-treated cells that were seeded onto glass coverslips and allowed to produce matrix for 48 h, following which the siRNA-treated cells were then stripped away, leaving behind CM. Untreated cells were seeded onto the CM and left to adhere for 3 h, and then immunocytochemistry for α5 integrin was carried out. The phenotype of long, fibrillar adhesions was repeated, indicating that α5 integrin changes occur in response to a modified matrix (Fig. 7A). Signalling was examined in a similar fashion: untreated cells were adhered to CM for 45, 90 and 180 min, then cells were lysed and signalling responses examined. Cells adhered to the conditioned matrix from ADAMTS-1 or syndecan-4 knockdown cells showed increased paxillin and ERK signalling, suggesting that the altered matrix is sufficient to induce the more migratory phenotype (Fig. 7B; Fig. S7).

Fibulin-1, a co-factor for ADAMTS-1 and a matrix protein that has been reported to inhibit migration, showed enhanced expression at 180 min post-adhesion to fibronectin in NTC-treated cells, but not ADAMTS-1- or syndecan-4 siRNA-treated cells (Fig. 7C; Fig. S8) (Lee et al., 2005; Twal et al., 2001). These data support a mechanism by which ADAMTS-1 and syndecan-4 regulate adhesion by inducing expression of the matrix protein fibulin-1.

## DISCUSSION

Our work provides new insights into a functional connection between ADAMTS-1 and the cell surface proteoglycan syndecan-4. Knockdown of either protein via siRNA results in similar responses with regard to angiogenesis, cell migration and integrin α5 trafficking. Our data support the notion that ADAMTS-1 is an inhibitor of angiogenesis, and we offer novel data that this occurs in co-ordination with syndecan-4.

Previous work has shown that ADAMTS-1 inhibits angiogenesis through direct sequestration of the major pro-angiogenic growth factor VEGF_165_, reducing its bioavailability (Luque et al., 2003). This has also been shown to be true for ADAMTS-4, which can bind VEGF and inhibit angiogenesis in a similar fashion to ADAMTS-1 (Hsu et al., 2012). The heparin binding domain of VEGF_165_ is necessary for its interaction with ADAMTS-1, and work by Iruela-Arispe et al. has suggested that this interaction may be reliant on a heparin-like molecule (Iruela-Arispe et al., 2003). One such candidate is syndecan-4, a heparan sulfate proteoglycan and the major syndecan in endothelial cells, known to interact with heparin-binding growth factors such as VEGFs, PDGFs and FGFs (Harburger and Calderwood, 2008). Thus, given the numerous connections between ADAMTS-1 and syndecan-4 and the dependence of ADAMTS-15 on syndecan-4 to enhance migration ability, we sought to investigate the relationship between syndecan-4 and ADAMTS-1.

Importantly, ADAMTS-1 has been reported to cleave the ectodomain of syndecan-4, promoting migration in a way that reflects genetic deletion of the proteoglycan (Rodríguez-Manzaneque et al., 2009). However, we were unable to detect evidence of a cleaved form of syndecan-4 released from the fibroblasts and endothelial cells used in our studies. Instead we were able to demonstrate a convergent functional relationship between ADAMTS-1 and syndecan-4 as ADAMTS-1 siRNA treatment caused a significant reduction in syndecan-4 display at the cell surface, indicating that syndecan-4 is dependent on ADAMTS-1. A key finding from our work is that this connection between ADAMTS-1 and cell surface expression of syndecan-4 involves matrix metalloproteinase-9 (MMP9).

Syndecan ectodomain shedding is an important regulatory mechanism allowing for rapid changes in cell surface receptor dynamics; shedding occurs juxtamembrane and can be carried out by a number of matrix proteinases (Manon-Jensen et al., 2010). MMP7 and MMP14 are both able to shed the extracellular domain of syndecan-2, and MMP9 has been shown to shed syndecan-4 from the cell surface in response to TNFα (Kwon et al., 2014; Lee et al., 2017; Ramnath et al., 2014). There was a significant increase in MMP9 expression in ADAMTS-1 siRNA-treated cells, and upon loss of MMP9, via either an inhibitor or siRNA depletion, syndecan-4 was no longer lost from the cell surface. We therefore propose that the loss of ADAMTS-1 results in increased shedding of syndecan-4 that is mediated by MMP9.

At this point, we are not able to identify the mechanism by which ADAMTS-1 knockdown results in increased MMP9 expression. One possibility, however, is that it occurs as a result of a shift in the angiogenic signalling balance in endothelial cells. ADAMTS-1 is anti-angiogenic, whereas MMP9 is highly pro-angiogenic, and can trigger the ‘angiogenic switch’, a process in tumours where the balance of pro- and anti-angiogenic factors swings towards a pro-angiogenic outcome (Bergers et al., 2000). MMP9 promotes release of VEGF bound in the matrix to heparan sulfate chains through cleavage (Hawinkels et al., 2008). VEGF induces MMP9 expression, which leads to elevated free VEGF levels resulting in a positive feedback loop (Hollborn et al., 2007). Similarly, VEGF can up-regulate ADAMTS-1 expression, suggesting a negative feedback mechanism at play (Xu et al., 2006). It seems possible that loss of ADAMTS-1 disturbs the fine balance of VEGF signalling, resulting in the cells switching to a more angiogenic phenotype, and the up-regulation of MMP9.

We investigated the possibility that syndecan-4 works co-operatively with ADAMTS-1 to inhibit angiogenesis through sequestration of VEGF. This idea contrasts with several hypotheses that syndecan-4 acts as a co-receptor for VEGF to enhance signalling, as is the case for FGFs. Through co-immunoprecipitations, we demonstrated that syndecan-4 does indeed bind VEGF. We interpret this binding as sequestration, as an increase in VEGF signalling and free VEGF is seen upon syndecan-4 depletion. These data strongly suggest that syndecan-4 does not promote VEGF signalling, but instead alongside ADAMTS-1 sequesters VEGF to inhibit angiogenesis. We further demonstrated this finding in a physiologically relevant *ex vivo* model of angiogenesis and observed increased microvessel sprouting in an aortic ring assay with ADAMTS-1 or syndecan-4 siRNA depletion. It therefore seems plausible that MMP9, ADAMTS-1 and syndecan-4 work together in a complex equilibrium to balance VEGF bioavailability and regulate angiogenesis.

For angiogenesis to occur, endothelial cells must proliferate and migrate – processes that are heavily influenced by VEGF signalling. Loss of ADAMTS-1 resulted in increased proliferation, although this result was not mirrored by loss of syndecan-4. This implies that the two molecules have distinct as well as shared actions. Syndecan-4 is a major signalling nexus involved as a partner in the actions of several growth factors as well as a source of intracellular signals itself. These multiple pathways could negate the impact of sequestration of VEGF by syndecan-4. Likewise, ADAMTS-1 functions independently of syndecan-4, specifically in relation to angiogenesis by releasing anti-angiogenic peptides from TSP-1 and TSP-2 through cleavage (Lee et al., 2006). At the organism level, although *Sdc4* and *Adamts1* null mice share a delayed wound healing response (Echtermeyer et al., 2001), the models do not phenocopy each other. Whilst *Sdc4* knockout mice are relatively healthy, *Adamts1* KO mice exhibit developmental issues with stunted growth and high embryonic mortality (Echtermeyer et al., 2001; Shindo et al., 2000).

Previous work on fibroblasts from *Sdc4*−/− animals showed that syndecan-4 was necessary for directional migration to occur (Bass et al., 2007). However, in our experiments, acute depletion of syndecan-4 or ADAMTS-1 in endothelial cells resulted in increased migration speed. This observation was only seen on fibronectin, with decreased or no change in migration speed on collagen. Fibronectin matrix-dependent migration effects have been noted previously with the ADAMTSs; work by Kelwick et al. showed that the closely related ADAMTS-15 was able to inhibit MDA-MB-231 cell migration on fibronectin matrices, and that this effect was dependent on syndecan-4 (Kelwick et al., 2015a). In endothelial cells, α5 integrin functions as the major fibronectin receptor. Interestingly, syndecan-4 regulates adhesion synergistically with fibronectin and α5β1 integrin (Morgan et al., 2007), as well as regulating integrin trafficking. Phosphorylation of syndecan-4 acts as a molecular switch, controlling levels of αVβ3 and α5β1 internalisation and recycling, and affecting cell behaviour (Morgan et al., 2013).

Due to the clear connection between syndecan-4, fibronectin and integrins, we sought to investigate α5 integrin as a potential mediator of migratory effects. Immunocytochemical and cell surface biotinylation data support an important role for syndecan-4 in regulation of α5 integrin trafficking. ADAMTS-1 knockdown also appeared to regulate α5 integrin trafficking, but whether this is a direct effect is unclear, although it would be consistent with the resulting loss of syndecan-4. Despite this, siRNA knockdowns of ADAMTS-1 and syndecan-4 repeated in α5 null endothelial cells maintained the phenotypes of increased VEGF signalling, increased free VEGF, faster random migration and larger focal adhesions, suggesting that α5 integrin is not necessary for ADAMTS-1 and syndecan-4 regulated angiogenesis and migration. Therefore, we hypothesised that knockdowns of ADAMTS-1 and syndecan-4 may result in ECM alterations, resulting in downstream changes to integrin trafficking. Both proteins interact with the ECM: ADAMTS-1 is secreted and anchors in the ECM, while syndecan-4 forms cell–ECM attachments (Gopal et al., 2017; Kuno and Matsushima, 1998). In support of this, non-treated naïve cells plated on ‘conditioned matrix’ generated by ADAMTS-1 and syndecan-4 siRNA-treated cells behaved in a similar manner to siRNA-treated cells, forming altered α5 adhesions, and displaying increased paxillin signalling.

Fibulin-1 is an ECM protein, and a co-factor for ADAMTS-1 (Lee et al., 2005); expression of fibulin-1 has been reported to result in a fibronectin-specific inhibition of migration in a cell type-dependent manner (Twal et al., 2001). In fibroblasts, this effect is dependent on syndecan-4 (Williams and Schwarzbauer, 2009). Fibulin-1 is indeed upregulated at 180 min post-adhesion in NTC-treated cells, but not in ADAMTS-1 or syndecan-4 siRNA cells. We therefore propose a mechanism for the control of migration in endothelial cells where ADAMTS-1 and syndecan-4 regulate production of fibulin-1, thereby suppressing migration and facilitating α5 integrin turnover.

This work builds on the current literature on ADAMTS-1 as an anti-angiogenic protein, demonstrating co-operation between ADAMTS-1 and the heparan sulfate proteoglycan syndecan-4. We also highlight a role for syndecan-4 in inhibiting migration and angiogenesis, which contrasts with some previous published data. Our studies therefore contrast with the work of Corti et al. (2019), who found that endothelial specific knockout of syndecan-2 in mice resulted in marked defects in angiogenesis, whereas knockout of syndecan-4 resulted in no such defects. This specificity was attributed to the enhanced 6-*O*-sulfation level in syndecan-2 versus syndecan-4 heparan sulfate (HS) chains, leading to superior levels of VEGFA_165_ binding by syndecan-2 versus syndecan-4 (Corti et al., 2019). Two points are relevant in attempting to understand data conflicts with regard to the role of syndecan-4 in angiogenesis and migration. Firstly, it is important to consider the model used. Much previous work has focused on the constitutive *Sdc4-*knockout mouse, and cells and tissues isolated from it (Echtermeyer et al., 2001). Compensatory mechanisms from developmental adaptation to loss of syndecan-4 are possible, and may affect phenotypes, as reported in a previous study (Bass et al., 2007). It is also now recognised that passenger mutations in knockout mice created using embryonic stem cells from the 129 strain may confound interpretation of data from these mice (Eisener-Dorman et al., 2009). Secondly, in our immortalised endothelial cell line syndecan-4 is the most highly expressed member of the syndecan family, therefore its effects may dominate. It seems likely therefore that both syndecan-2 and syndecan-4 may contribute to VEGFA_165_ binding and angiogenesis.

There is also a potential argument that previous migration studies had used fibroblasts derived from *Sdc4*-knockout mice, which may be differently affected by loss of syndecan-4 than the VEGF-responsive endothelial cells that are the focus of our work, due to the involvement of syndecan-4 in multiple growth factor signalling pathways. However, we have seen similar effects of siRNA-mediated knockdown of syndecan-4 on migration in fibroblasts as in endothelial cells, suggesting that these are not dependent on cell type. Our findings are lent support by work by Cavalheiro et al., who found increased migration speed when using short hairpin RNA (shRNA) to deplete syndecan-4 in endothelial cells (Cavalheiro et al., 2017). In future, the field could be greatly advanced by development of an endothelial specific syndecan-4 conditional knockout mouse that would aid in resolving issues of redundancy and compensation.

**Fig. 8.**
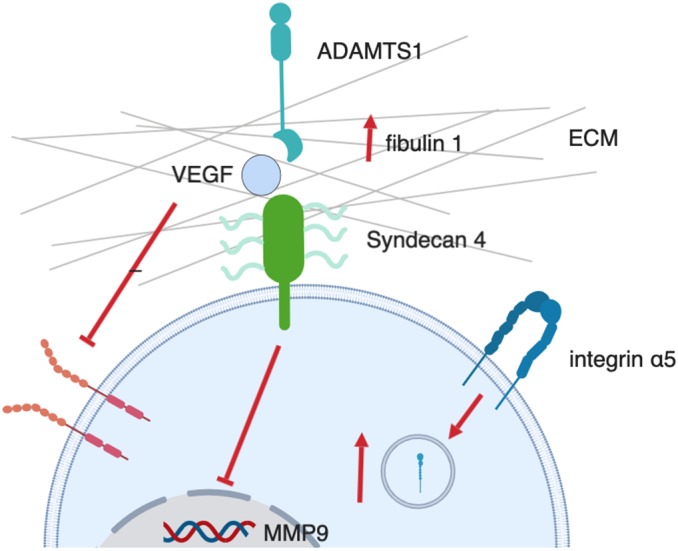
**Proposed model of interaction between ADAMTS-1 and syndecan-4.** Schematic outlining the interactions and functions of ADAMTS-1 and syndecan-4 in endothelial cells. Figure created using BioRender.

In conclusion, our work shows that ADAMTS-1 regulates cell surface expression of syndecan-4, and in endothelial cells both molecules act via sequestration of VEGF and inhibition of angiogenesis. ADAMTS-1 influences syndecan-4 levels on the cell surface via regulation of MMP9 production. Our studies lead to a proposed model of convergent roles for ADAMTS-1 and syndecan-4 in regulating the ECM, inhibiting migration, modulating integrin adhesions and activating the matrix protein fibulin-1 (Fig. 8).

## MATERIALS AND METHODS

### Animals

All animal experiments were performed in accordance with UK Home Office regulations and the European Legal Framework for the Protection of Animals used for Scientific Purposes (European Directive 86/609/EEC).

### Cell culture and isolation

Human umbilical vein endothelial cells (HUVECs) were purchased from Lonza (Basel, Switzerland), and cultured in EBM-2 medium supplemented with the SingleQuots kit (Lonza). 3T3 fibroblasts were cultured in high-glucose Dulbecco's modified Eagle's medium (DMEM; Invitrogen) supplemented with 10% fetal bovine serum (FBS) (HyClone, Invitrogen), 100 units/ml penicillin/streptomycin (Invitrogen), and 2 mM GlutaMAX (Invitrogen).

Primary mouse lung endothelial cells were isolated from adult mice as previously described by Reynolds and Hodivala-Dilke (2006). For immortalisation, ECs were treated with polyoma-middle-T-antigen (PyMT) retroviral transfection, as described by Robinson et al. (2009). Immortalised mouse lung endothelial cells (ECs) were cultured in IMMLEC medium, a 1:1 mix of Ham's F-12–DMEM medium (low glucose) supplemented with 10% FBS, 100 units/ml penicillin/streptomycin, 2 mM GlutaMAX and 50 μg/ml heparin (Sigma).

All cells were cultured at 37°C in a humidified chamber with 5% CO_2_. Cells were routinely screened for mycoplasma contamination. For experimental analyses, plates and flasks were coated with either human plasma fibronectin (FN; Millipore) or collagen I (Col I; Fisher Scientific, Loughborough, UK) overnight at 4°C. VEGF-A 164 (henceforth referred to as VEGF) was made in-house according to the method published by Krilleke et al. (2007).

### RNAi and lentiviral transfections

For siRNA transfections, ECs were transfected with 50 nM siRNA (Thermo Fisher Scientific) using the Amaxa nucleofector system (Lonza) according to the manufacturer's instructions, with a final siRNA concentration of 50 nM, and the nucleofection program T-005. For syndecan-4 (SDC4) ICC and ELISA, ECs were transfected with HA-tagged SDC4 construct (a generous gift from Dr. James Whiteford, Barts and the London School of Medicine and Dentistry, Queen Mary University of London, London, UK). Transfected cells were selected using a GFP reporter tag.

For shRNA knockdown of syndecan-4 and ADAMTS-1 in HUVECs, packaging plasmids (Addgene, #12260 and #12259) were mixed with shRNA plasmid (Mission shRNA, Sigma-Aldrich) in Optimem medium (Invitrogen) and Lipofectamine 2000 (Invitrogen) with the following ratios: 750 ng psPAX2, 250 ng pMD2.G and 1 μg shRNA. The mixture was transferred to 80% confluent 293T cells in 10 cm dishes for 6 h. The medium was replaced with regular DMEM 10% FBS and collected after 48 h. Medium containing virus was filtered through a 0.45 μm filter and used for HUVEC transduction with the addition of 8 μg/ml polybrene (Sigma). The target sequences used were as follows: human *Sdc4* sequence: 5′-CCGGCCTGATCCTACTGCTCATGTACTCGAGTACATGAGCAGTAGGATCAGGTTTTTG-3′. human *Adamts1* sequence: 5′-CCGGCCACAGGAACTGGAAGCATAACTCGAGTTATGCTTCCAGTTCCTGTGGTTTTTG-3′.

### Flow cytometry

For flow cytometric analysis, cells were removed from culture plates using citric saline buffer (1.35 M KCl, 0.15 M Na_3_C_6_H_5_O_7_). Cells were collected by centrifugation and resuspended in fluorescence-activated cell sorting (FACS) buffer [5% FBS in phosphate-buffered saline (PBS)] and labelled with anti-mouse syndecan-4 (KY/8.2, BD Biosciences, Allschwil, Switzerland), or isotype control (mouse IgG2a, Invitrogen) for 1 h. Cells were washed, resuspended in FACS buffer and incubated with fluorophore-conjugated secondary antibody (eBioscience, San Diego, CA, USA). Data were collected using a Beckman CytoFLEX flow cytometer and analysed using FlowJo.

For treatment with MMP inhibitors one of: 5 μM BB-94 (Abcam, Cambridge, UK), 10 μM CT1746 (CellTech, Slough, UK), 10 μM GM-6001 (Millipore) in 10 μl DMSO, or DMSO vehicle control, was added to cells for 18 h prior to flow cytometric analysis.

### RNA extraction, RT-PCR and real-time quantitative RT-PCR analyses

Total cell RNA was extracted using the SV Total RNA Isolation Kit (Promega) according to the manufacturer's instructions. RNA samples were reverse transcribed using the GoScript Reverse Transcriptase system (Promega). Quantitative real-time TaqMan PCR was carried out as described previously (López-Otín and Matrisian, 2007).

### Immunocytochemistry

siRNA-transfected ECs were seeded at a density of 2×10^5^ cells per well in 24-well plates on acid-washed and oven-sterilised glass coverslips pre-coated with FN or Col I. Cells were fixed at indicated time points in 4% paraformaldehyde, washed in PBS, blocked and permeabilised with 0.3% Triton X-100, 10% serum, and incubated with primary antibody diluted 1:100 in PBS for 1 h at room temperature (RT). Primary antibodies were: anti-HA tag (1:100; 2-2.2.14; Thermo Fisher Scientific), anti-paxillin (ab32084; Abcam) and anti-α5 integrin (1:100; ab150361; Abcam). Coverslips were washed with PBS, and incubated with the relevant Alexa-Fluor-conjugated secondary antibody (Invitrogen) diluted 1:500 in PBS for 45 min at RT. Coverslips were washed in PBS again, before mounting on slides with ProLong Gold containing DAPI (Invitrogen). Focal adhesion area calculations were carried out using Fiji software.

### Internalisation and recycling assays

#### Internalisation

siRNA transfected cells (1×10^6^) were seeded in 10 cm dishes. After adhering overnight, cells were serum starved for 3 h, transferred to ice, washed twice in cold PBS, and surface labelled at 4°C with 0.3 mg/ml NHS-SS-biotin (Pierce) for 30 min. One dish was collected prior to internalisation, to allow comparison of internalised protein versus total cell surface. Labelled cells were washed in cold PBS and transferred to IMMLEC at 37°C to allow internalisation. At each time point, medium was removed, dishes were transferred to ice and washed twice with ice-cold PBS. Biotin was removed from proteins remaining at the cell surface by incubation with the membrane-impermeable reducing agent MesNa (20 mM MesNa in 50 mM Tris–HCl; pH 8.6) for 1 h at 4°C. MesNa was quenched by the addition of 20 mM iodoacetamide (IAA) for 10 min. Cells were lysed in 200 mM NaCl, 75 mM Tris, 15 mM NaF, 1.5 mM Na_3_VO_4_, 7.5 mM EDTA, 7.5 mM EGTA and 1.5% Triton X-100, supplemented with protease inhibitor (Merck). Lysates were cleared by centrifugation at 10,000 ***g*** for 10 min. Levels of syndecan-4 internalisation were determined by capture ELISA. For α5 integrin internalisation analysis, biotinylated protein was isolated by immunoprecipitation using anti-biotin (Jackson ImmunoResearch), and levels of α5 integrin assessed by SDS-PAGE.

#### Recycling

After surface labelling, cells were incubated in IMMLEC at 37°C for 20 min to allow internalisation. After internalisation a plate was collected to allow quantification of recycled protein versus total internalised. Following removal of biotin from surface proteins using MesNA, the internalised fraction was then allowed to recycle to the membrane by returning cells to 37°C in IMMLEC. At the indicated times, cells were returned to ice and biotin was removed from recycled proteins by a second reduction with MesNa. Biotinylated syndecan-4 was then determined by capture-ELISA.

#### Capture-ELISA

Microplates (96-well; R&D Systems) were coated overnight with 5 μg/ml HA-tag antibody (1:1000; 51064, Proteintech) in PBS at RT. The plates were blocked in PBS containing 0.05% Tween-20 (0.05% PBS-T) with 1% BSA for 1 h at RT. HA-SDC4 was captured by 2 h incubation of 100 μl cell lysate (1 μg/μl) at RT. Unbound material was removed by extensive washing with 0.05% PBS-T. Wells were incubated with streptavidin-conjugated horseradish peroxidase (R&D Systems) in 0.05% PBS-T containing 1% BSA for 1 h at RT. Following further washing, biotinylated SCD4 was detected with tetramethylbenzidine (R&D Systems).

### Zymography

Zymography was performed using SDS-PAGE (7.5%) gels co-polymerised with 1 mg/ml gelatin. Protein concentrations of media samples were equalised, and added to 5× non-reducing sample buffer (Thermo Fisher Scientific). After electrophoresis, gels were washed twice for 30 min in wash buffer (2.5% Triton X-100, 50 mM Tris–HCl, 5 mM CaCl_2_, 1 μM ZnCl_2_) at RT. Gels were incubated overnight in incubation buffer (1% Triton X-100, 50 mM Tris–HCl, 5 mM CaCl_2_, 1 µM ZnCl_2_) at 37°C. Gels were stained in Coomassie Blue R 250 (Thermo Fisher Scientific) in a mixture of methanol:acetic acid:water (4:1:5) for 1 h and destained in the same solution without dye. Gelatinase activities were visualised as distinct bands.

### Western blot analysis

ECs were seeded at 2.5×10^5^ cells per well in 6-well plates coated with 10 μg/ml FN. After 24 h, cells were starved for 3 h in serum-free medium (Opti-MEM; Invitrogen). VEGF was then added to a final concentration of 30 ng/ml. Cells were lysed at the indicated times (Fig. 3) in radioimmunoprecipitation assay (RIPA) buffer [25 mM Tris-HCl, pH 7.5, 150 mM NaCl, 0.1% SDS, 0.5% sodium deoxycholate, 1% Triton X-100, supplemented with protease inhibitor (Merck)]. After protein quantification using the DC Bio-Rad assay, 30 μg of protein from each sample was loaded onto 8% polyacrylamide gels. For paxillin analysis, samples were loaded onto a 4–12% gradient gel for better resolution. The protein was transferred to a nitrocellulose membrane and incubated for 1 h in 5% milk powder and PBS plus 0.1% Tween-20 (0.1% PBS-T), followed by an overnight incubation in primary antibody diluted 1:1000 in 5% bovine serum albumin (BSA) and 0.1% PBS-T at 4°C. The blots were then washed three times with 0.1% PBS-T and incubated with the relevant horseradish peroxidase (HRP)-conjugated secondary antibody (Dako, Santa Clara, CA, USA) diluted 1:2000 in 5% milk and PBS-T, for 1 h at room temperature. Chemiluminescence was detected on a Bio-Rad Gel Doc XR+ (Bio-Rad).

Antibodies (all used at 1:1000 and purchased from Cell Signaling Technology, unless noted otherwise) were: anti-phospho (Y1175) VEGFR2 (clone 19A10); anti-VEGFR-2 (clone 55B11); anti-phospho (Thr202/Tyr204) p44/42 MAPK Erk1/2 (clone D13.14.4E); anti-total p44/42 MAPK Erk1/2, anti-HSC70 (clone B-6, Santa Cruz Biotechnology), anti-human syndecan-4 (ab24511, Abcam), anti-phospho (Tyr118) paxillin (2541), anti-phospho (Tyr925) focal adhesion kinase (FAK), anti-FAK and anti-GAPDH (6C5, Abcam).

Fiji software (https://imagej.net/Fiji) was used for quantification of band densities.

### Immunoprecipitation assay

HUVECs were grown to 80–90% confluency in 10 cm dishes coated with 10 μg/ml FN in PBS. Cells were lysed in RIPA buffer. Four hundred micrograms of total protein from each sample was immunoprecipitated by incubation with protein G Dynabeads (Invitrogen) coupled to 1 μg mouse-anti-human VEGF-A antibody (VG-1, Abcam) on a rotator overnight at 4°C. Immunoprecipitated complexes were washed three times with 0.2 ml of RIPA buffer, and once in PBS, before being added to, and boiled in NuPAGE sample reducing agent and sample buffer (Life Technologies) for western blotting.

### VEGF ELISA

For quantification of free VEGF present in the media, ECs were plated at a density of 2.5×10^5^ cells per well of a 6-well plate. Cells were chilled to 4°C, medium was removed and cells were washed twice in ice-cold PBS. Medium was replaced with ice-cold Opti-MEM containing VEGF at a final concentration of 30 ng/ml. Media were diluted 1:10 in 1% BSA in PBS, and VEGF concentration was quantified using a mouse VEGF duo-set ELISA, according to the manufacturer's instructions (DY493, R&D Systems).

### Proliferation assay

siRNA-transfected ECs were seeded onto 10 μg/ml FN-coated glass coverslips (1.5×10^4^ cells per well of a 24-well plate). After 4 h, the medium was replaced with IMMLEC containing 10 nM BrdU. After 12 h cells were fixed in 4% paraformaldehyde. To stain, cells were incubated in 1 M HCl for 30 min at RT, then permeabilised with PBS 0.25% Triton X-100 for 10 min and blocked by a 20 min incubation in Dako block (Agilent). BrdU was detected by incubation with anti-BrdU (ICR1, Abcam) diluted 1:100 in PBS for 1 h at room temperature. Coverslips were washed in PBS, then incubated with anti-sheep Alexa-Fluor (Invitrogen) for 1 h at RT. After further PBS washes, coverslips were mounted in ProLong Gold containing DAPI (Invitrogen).

### Random migration assay

siRNA-transfected ECs were trypsinised and seeded at 1.5×10^4^ cells per well in 24-well plates coated with 10 μg/ml FN or 10 μg/ml Col I in PBS, and allowed to adhere overnight. The medium was then replaced with fresh IMMLEC. One phase contrast image per well was taken live every 10 min in a fixed field of view using an inverted Axiovert microscope (Zeiss) for 16 h at 37°C and 5% CO_2_. Individual cells were then manually tracked using the Fiji cell tracking plug-in, mTrackJ (https://imagej.net/MTrackJ) and the speed of random migration was calculated in nanometres moved per second.

### *Ex vivo* aortic ring assay

Thoracic aortae were isolated from 6- to 8-week-old adult C57BL/6 mice and prepared for culture as described extensively by Baker et al. (2012). Syndecan-4 or ADAMTS-1 depletion was induced using 1 μM siRNA and oligofectamine. Where indicated, VEGF was added at 30 ng/ml. Microvessel growth of aortic rings was quantified after 6–10 days.

### Conditioned matrix generation

For experiments using conditioned matrix (CM), siRNA-treated cells were seeded at ∼70% confluence and allowed to produce matrix for 48 h. Plates were washed in PBS and cells were removed by incubation in 20 mM ammonium hydroxide. After extensive washing in PBS, untreated cells could then be seeded onto the CM.

### Statistics

Statistical analysis was conducted in R. Graphing was conducted in R or GraphPad. Where data were normally distributed with equal variance, a Student's *t*-test was used to determine statistically significant differences between conditions. Normal distributions were tested for using a Shapiro–Wilk test. Where data did not fit the normal distribution (focal adhesion sizes) a Kruskal–Wallis non-parametric test was used to determine statistical significance. Bar charts represent the mean and the standard error of the mean (s.e.m.), unless otherwise stated. Asterisks represent *P*-values as follows: *P<0.05, **P<0.01, ***P<0.001 and ****P<0.0001.

## Supplementary Material

Supplementary information
