# Supplementary material for: ADAMTS-1 and syndecan-4 intersect in the regulation of cell migration and angiogenesis
Source: J Cell Sci. 2020 Apr 8;133(7):jcs235762. doi: 10.1242/jcs.235762 (PMC7157938; doi:10.1242/jcs.235762)
Supplement: Supplementary information [file joces-133-235762-s1.pdf]

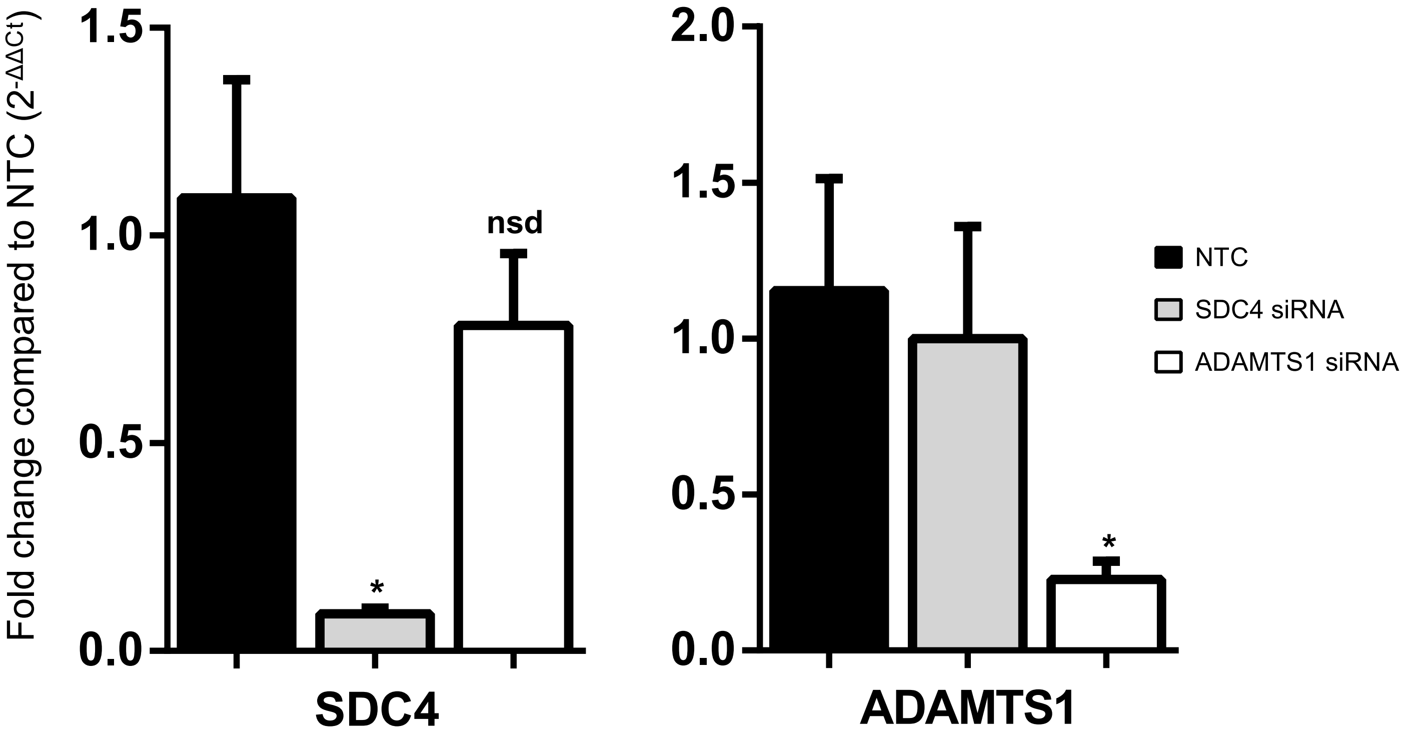

Figure S1: SiRNA successfully depletes targets in endothelial cell lines. Taqman qPCR of Ecs treated with ADAMTS-1 or SDC4 siRNA showing relative levels of *Sdc4* and *Adamts-1* in 4 independent experiments, mean±S.E.M, *p<0.005 compared to control cells by Student's t-test

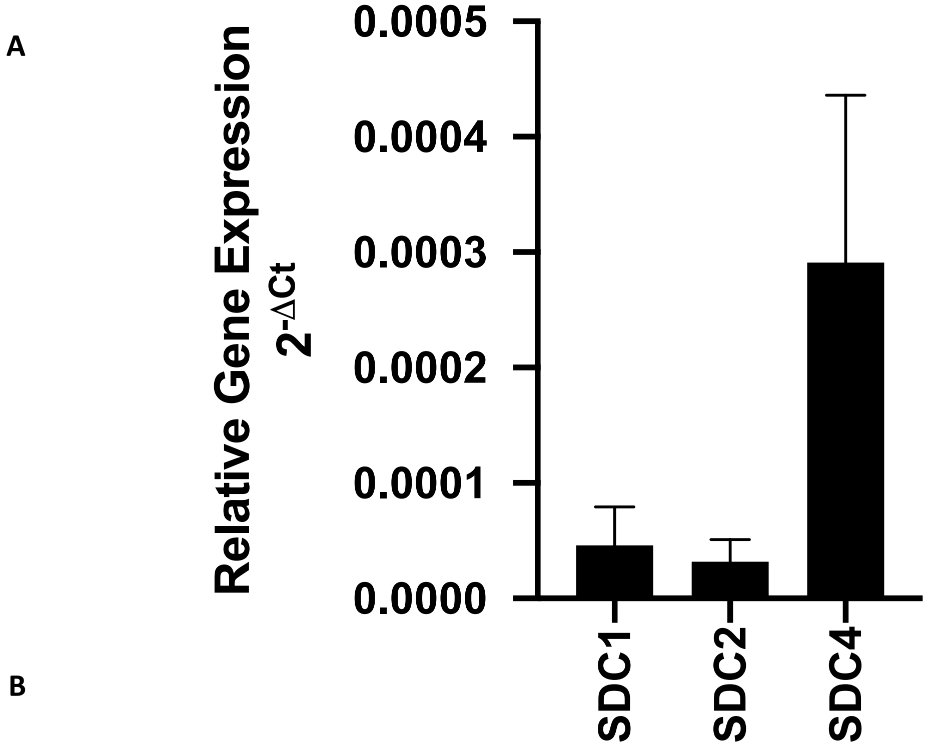

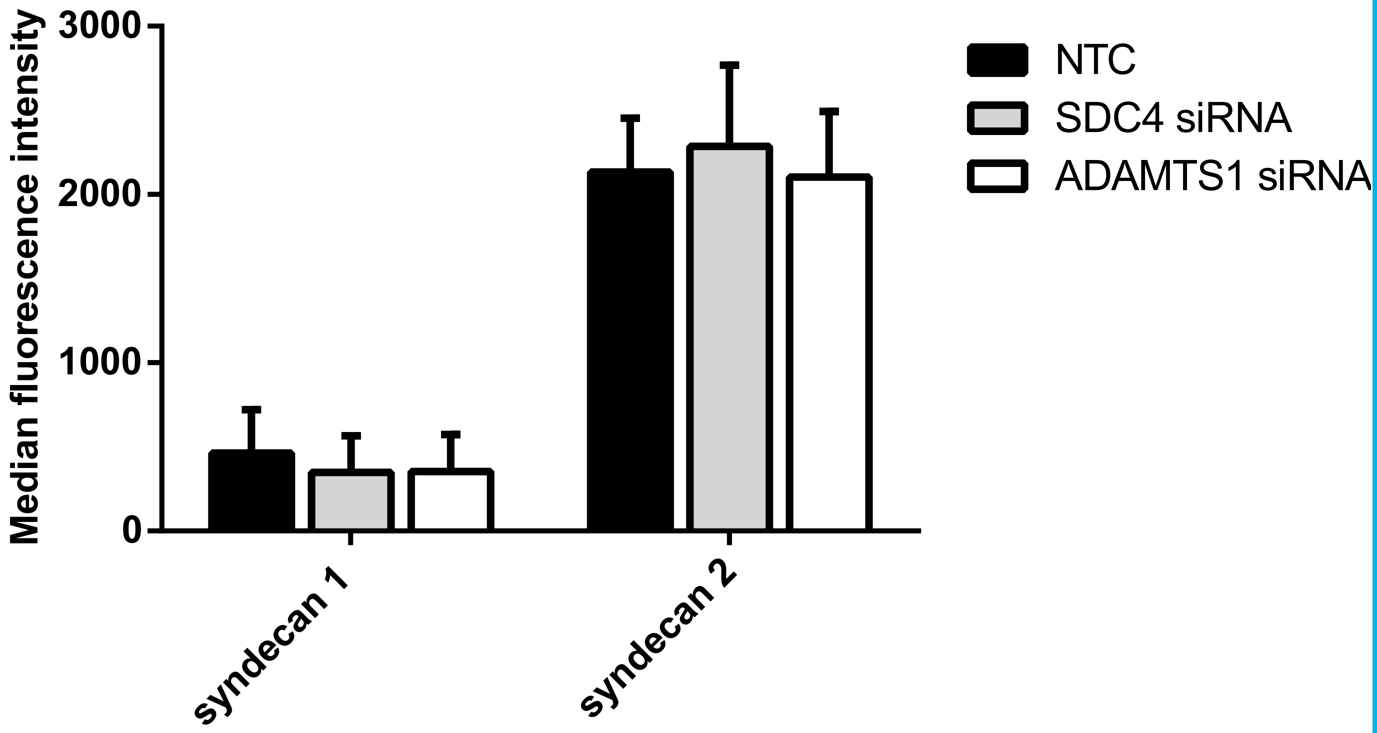

Figure S2: Syndecan 1 and 2 are unaffected by ADAMTS-1 or syndecan 4 siRNA depletion. A) TaqMan qPCR demonstrating levels of syndecan 1 (SDC1), 2 (SDC2) and 4 (SDC4) in RNA isolated from untreated immortalised endothelial cells. Bar chart shows relative expression, normalised to a housekeeping control (18s) (N=3). B) Flow cytometric analysis of syndecan 1 and 2 surface levels on ECs treated with NTC, SDC4, or ADAMTS-1 siRNA. The graph shows mean median fluorescence intensity values. Values were calculated after gating on forward and side scatter and normalised to isotype controls. N = 3 independent experiments. Charts show mean±S.E.M.

Journal of Cell Science • Supplementary information

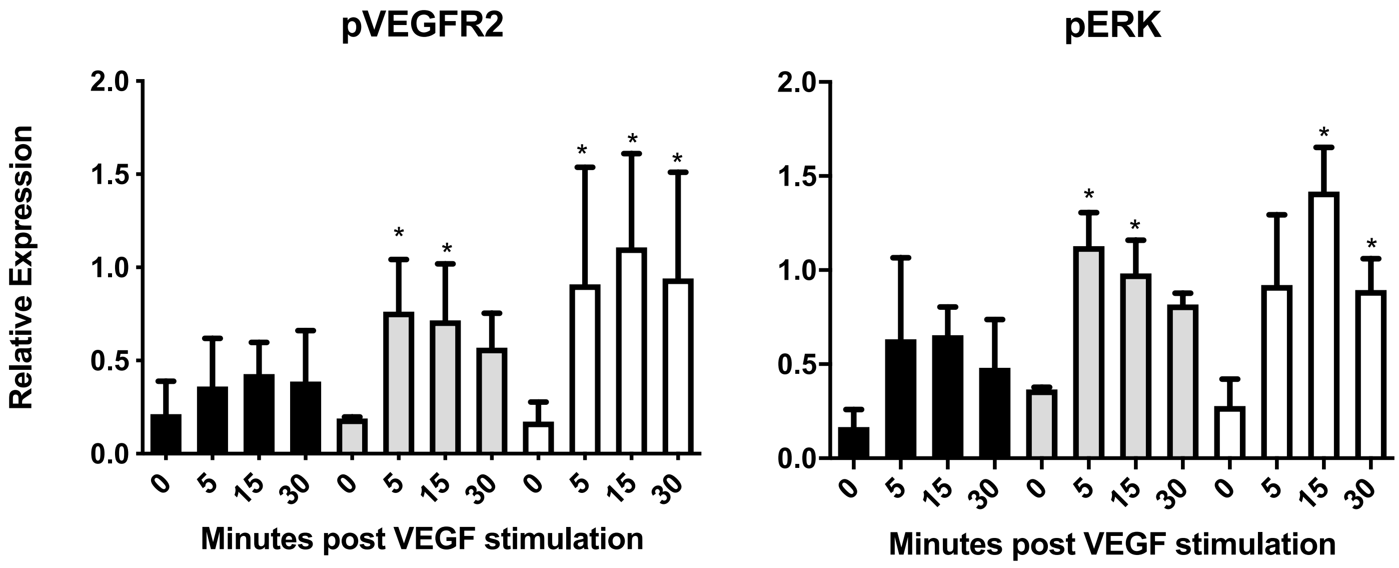

Figure S3: VEGF signalling is increased upon depletion of ADAMTS-1 or SDC4. Image J densitometric quantification of VEGF timecourses (figure 3A). Phosphorylated protein expression levels were calculated relative to total protein. (N=3 independent experiments, charts show mean±S.E.M, *P<0.05 calculated using a Student's T test compared to timepoint control).

Journal of Cell Science • Supplementary information

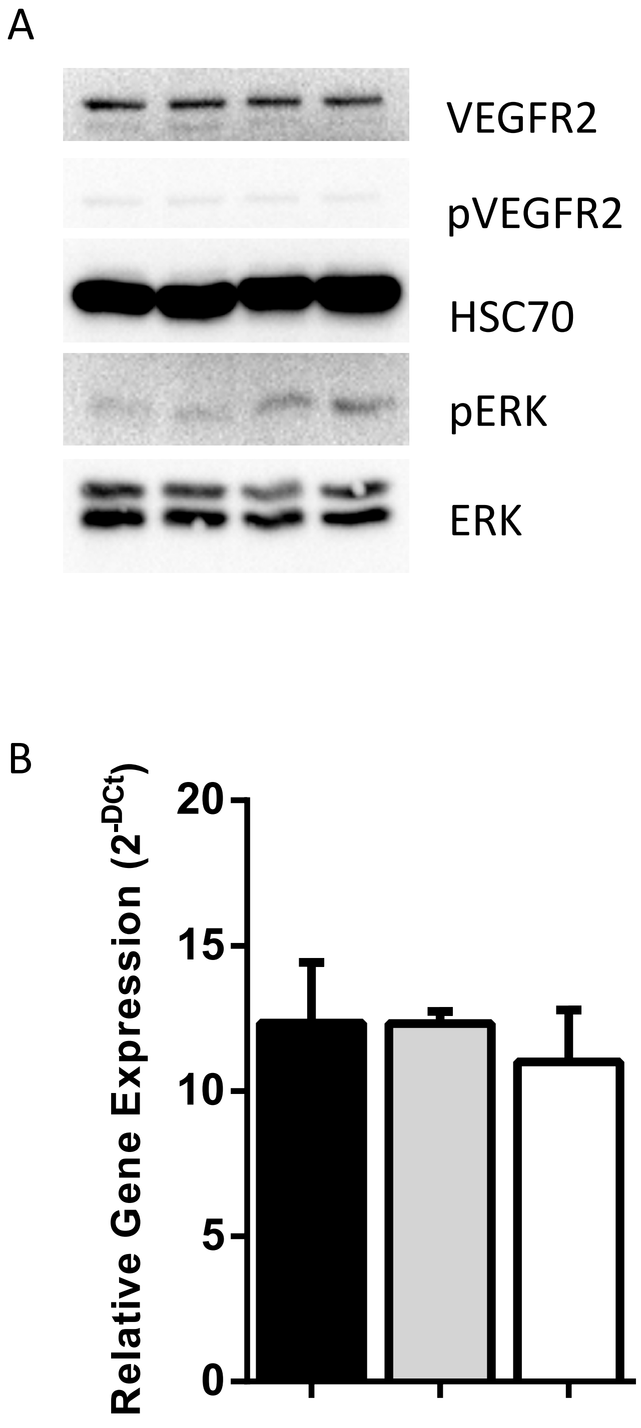

Figure S4: **Increased free VEGF in media is not due to increased transcription or altered signalling. A)** 30 ng/µl $VEGFA_{164}$ was added to ECs at 4ºC, after 30 minutes cells were lysed and western blotted for VEGFR2, ERK and their phosphorylated forms. **B)** RNA was collected 48 post siRNA transfection of ECs, qPCR was performed to determine VEGF expression levels. Bars represent mean±S.E.M, N=3.

Journal of Cell Science • Supplementary information

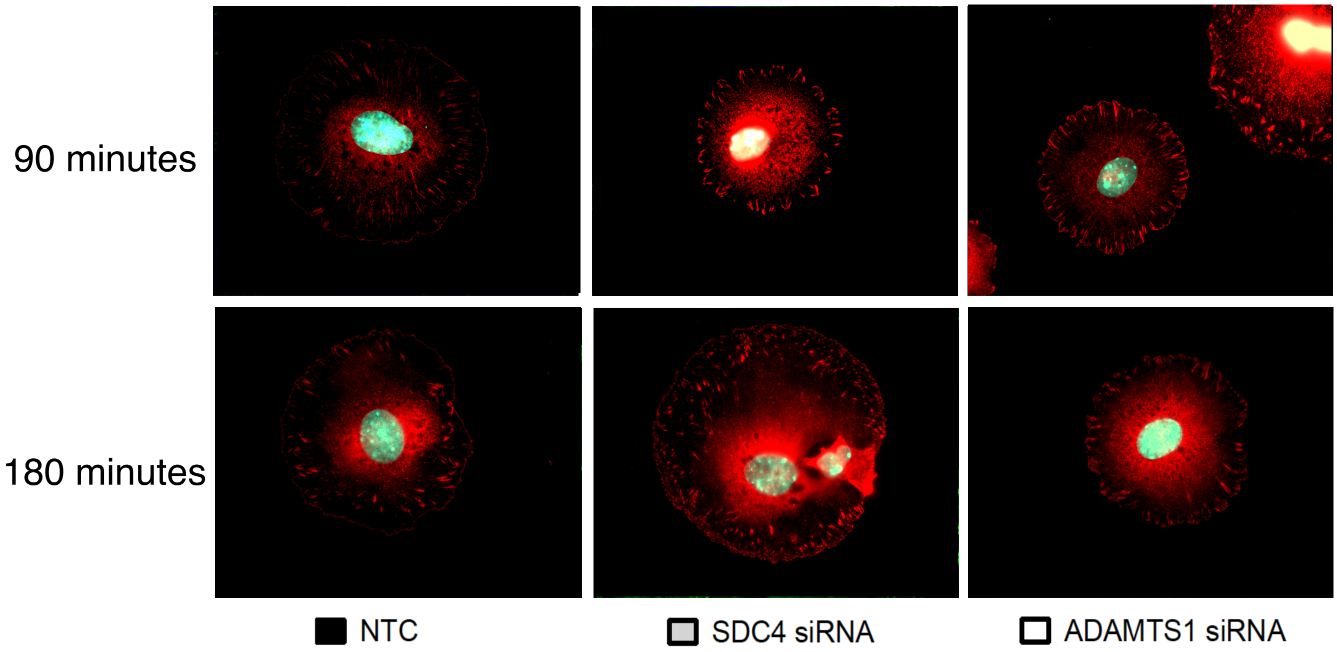

Figure S5: **ADAMTS-1 or SDC4 depletion does not affect adhesion to collagen**. Representative images of siRNA-treated ECs adhered to collagen coated glass coverslips for 90 or 180 minutes. Cells fixed and stained for focal adhesions with paxillin (*green*) and the nucleus with DAPI (*blue*). N =3 independent experiments. Scale bar = 10 µm.

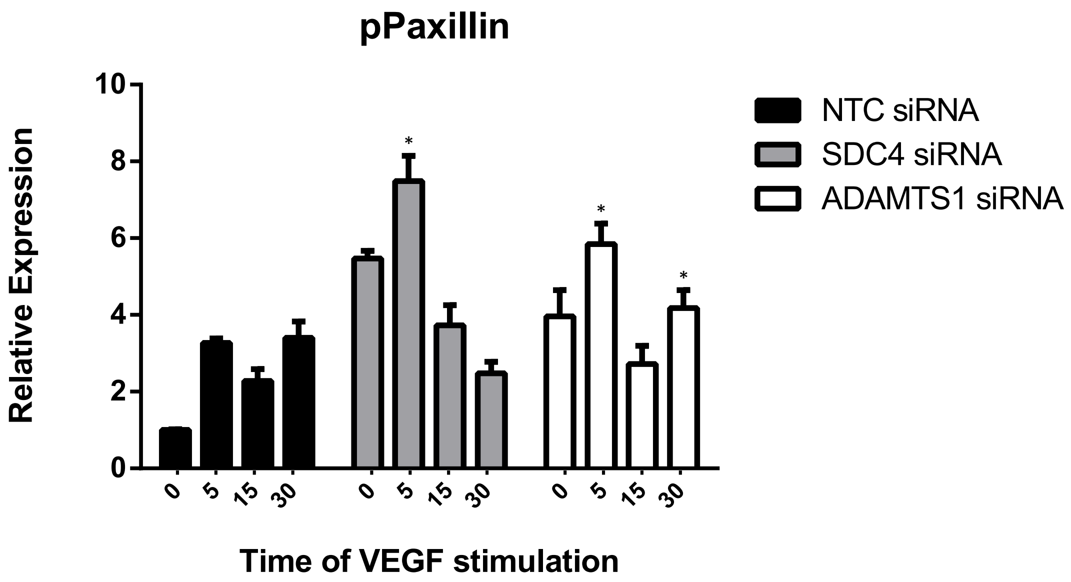

Figure S6: ADAMTS-1 or SDC4 depletion increases paxillin signalling in response to VEGFA$_{164}$. Image J densitometric quantification of VEGF timecourses (figure 5C). Phosphorylated paxillin expression level was calculated relative to total protein, *P<0.05 calculated using a Student's T test compared to timepoint control (N=3).

Journal of Cell Science • Supplementary information

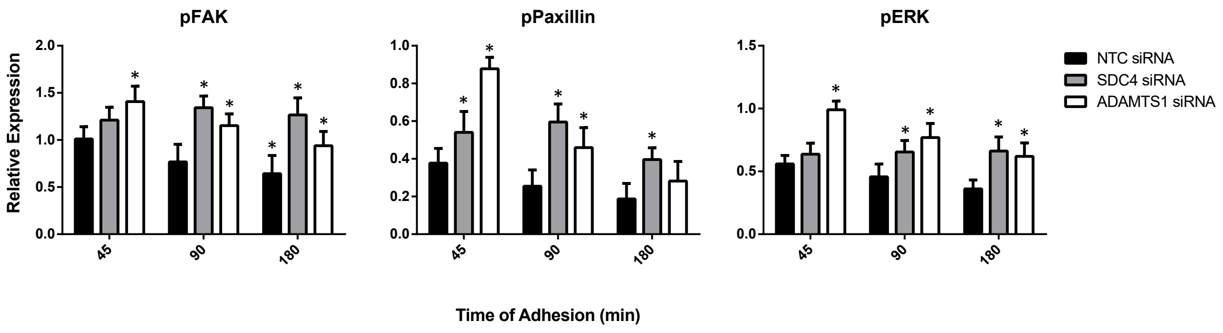

Figure S7: untreated cells show increased signalling responses to conditioned matrix. Image J densitometric quantification conditioned matrix timecourse (figure 5C). Phosphorylated protein expression levels were calculated relative to total protein, *P<0.05 calculated using a Student's T test compared to timepoint control). (N=3).

Journal of Cell Science • Supplementary information

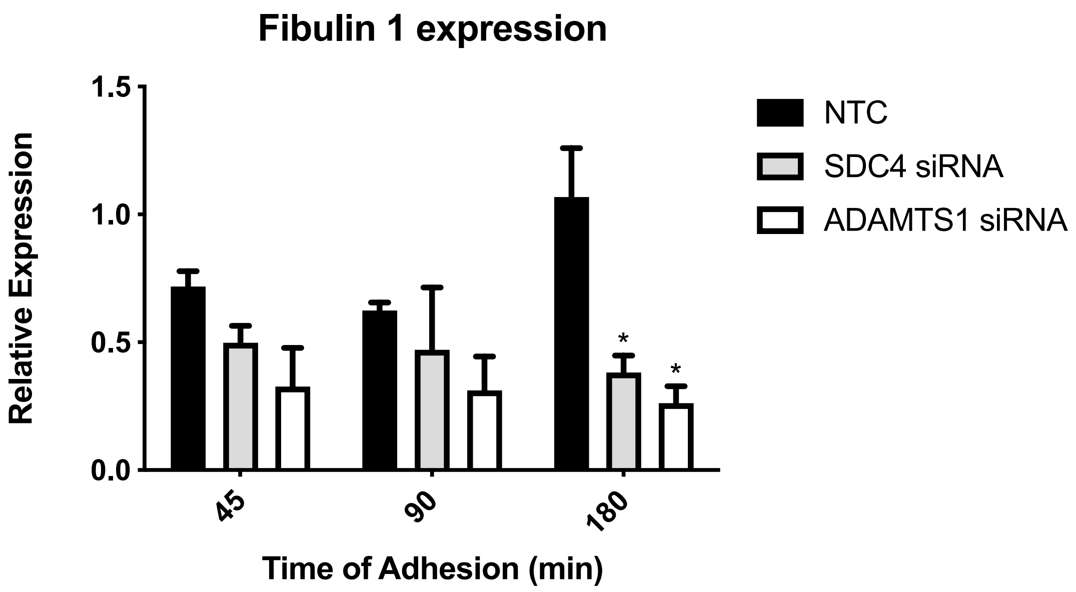

Figure S8: NTC cells, but not ADAMTS-1 or SDC4 siRNA treated cells, express increased fibulin 1. Image J densitometric quantification of fibronectin adherence timecourses (figure 7C). Fibulin 1 expression was calculated relative to GAPDH loading control. Bars represent mean±S.E.M, *P<0.05 calculated using a Student's T test (N= 3 independent experiments).
